# Differential Beta and Gamma Activity Modulation during Unimanual and Bimanual Motor Learning

**DOI:** 10.1523/JNEUROSCI.2187-24.2025

**Published:** 2025-04-29

**Authors:** Min Wu, Marleen J. Schoenfeld, Carl Lindersson, Sven Braeutigam, Catharina Zich, Charlotte J. Stagg

**Affiliations:** ^1^Wellcome Centre for Integrative Neuroimaging, FMRIB, Nuffield Department of Clinical Neurosciences, University of Oxford, Oxford OX3 9DU, United Kingdom; ^2^Medical Research Council Brain Network Dynamics Unit, Nuffield Department of Clinical Neuroscience, University of Oxford, Oxford OX1 3TH, United Kingdom; ^3^Department of Neurophysiology and Pathophysiology, University Medical Center Hamburg-Eppendorf, Hamburg 20246, Germany; ^4^Medical Sciences Division, Oxford Centre for Human Brain Activity, University of Oxford, Oxford OX3 7JX, United Kingdom; ^5^Department of Clinical and Movement Neuroscience, UCL Queen Square Institute of Neurology, London WC1N 3BG, United Kingdom

**Keywords:** beta ERD, beta ERS, bimanual movement, gamma ERS, motor learning

## Abstract

Movement-related dynamics in the beta and gamma bands have been studied in relation to motor execution and learning during unimanual movements, but their roles in complex bimanual tasks remain largely unexplored. This study aimed to investigate how beta and gamma activity differs between unimanual and bimanual movements and how these neural signatures evolve during the learning process. Our motor task incorporated varying levels of bimanual interaction: unimanual, bimanual-equal, and bimanual-unequal. Magnetoencephalography data were recorded in healthy participants (*N* = 43, 27 females) during task performance, and beta and gamma activity was quantified. As expected, increasing task complexity from unimanual to bimanual-equal and then to bimanual-unequal movements resulted in slower and less accurate performance. Across all conditions, significant beta event-related desynchronization (ERD) and gamma event-related synchronization (ERS) were observed during movement, as well as beta ERS after movement. Bimanual movements exhibited greater beta ERD, beta ERS, and gamma ERS compared with unimanual movements. With practice, participants demonstrated faster and more accurate movements, accompanied by enhanced beta ERS responses. Furthermore, learning-related reductions in errors correlated with increases in beta ERS. These findings suggest the distinct behavioral and neural demands of unimanual versus bimanual movements and highlight the important role of beta activity in motor performance and learning.

## Significance Statement

Bimanual movements, which dominate daily motor behaviors, require finely tuned coordination between the two hands yet remain poorly understood at the neurophysiological level. Using magnetoencephalography, we tested neural responses to a novel movement task incorporating varying levels of bimanual interaction. We demonstrate that greater task complexity elicits enhanced movement-related brain activity in the beta and gamma frequency bands. Motor learning is associated with an increase in beta movement-related synchronization that correlates with improved movement accuracy. This study provides novel insights into how beta and gamma brain activity adapt to increasing movement complexity and motor learning.

## Introduction

Complex bimanual movements requiring a finely tuned coordination between the upper limbs are essential for daily activities. However, despite their ubiquity, research into the neural mechanisms underlying motor function has predominantly focused on unimanual motor tasks (Gonzalez Castro et al., 2014; Dekleva et al., 2018; Alhussein and Smith, 2021) or simple bimanual motor tasks involving only finger actions (Mechsner et al., 2001; Liuzzi et al., 2011; Brandes et al., 2017). This focus extends to research on motor skill acquisition, which has traditionally prioritized unimanual movements over more complex bimanual movements (Andres and Gerloff, 1999). However, investigating complex bimanual movements that more accurately reflect real-life motor function reveals rich insights into the neural mechanisms of motor control and learning.

Behavioral and neurophysiological studies have revealed important distinctions between unimanual and bimanual movements, as well as differences between bimanual tasks. Unimanual movements are generally performed with greater precision and ease than bimanual movements (Sisti et al., 2011). Bimanual movements require greater interhemispheric cooperation, with efficient communication between the motor cortices becoming increasingly crucial as task complexity increases (Serrien, 2008; Rueda-Delgado et al., 2017). Many studies on bimanual movements have predominantly focused on interhemispheric coupling (Andres et al., 1999; Serrien and Brown, 2002; Serrien et al., 2003; Serrien, 2008). However, beyond this, local brain activity in the beta band (13–30 Hz) plays a central role in movement, showing a significant power decrease during movement execution [the event-related desynchronization (ERD)] and a power increase following movement cessation [the event-related synchronization (ERS); Pfurtscheller and Aranibar, 1977; Pfurtscheller et al., 1997b]. The beta ERD is hypothesized to be related to task complexity; however, studies report contradictory findings (Gross et al., 2005; Houweling et al., 2008, 2010; Rudisch et al., 2023).

Movement-related beta activity is also postulated to have a role in the acquisition of motor skills. In simple reaching tasks, a practice-related strengthening of the beta ERD, the beta ERS, and the modulation depth (i.e., the difference between ERD and ERS) has been observed (Tan et al., 2014b; Nelson et al., 2017; Ricci et al., 2019). In bimanual tasks, Boonstra et al. (2007) reported overall beta power modulation during task learning but did not distinguish between beta ERD and ERS, leaving the specific roles of beta ERD and ERS in bimanual motor learning unclear. Moreover, these studies, primarily focused on unimanual movements or discrete bimanual movements (e.g., finger tapping or simple flexion), limiting the generalization of their results to more complex continuous bimanual movements, which constitute most human motor activities in daily life.

There is also a growing interest in the role of mid-gamma activity (60–90 Hz) in motor control and motor learning (Nowak et al., 2018; Zich et al., 2021). Gamma is a prokinetic emergent property of the motor cortices and increases in power during motor execution—the gamma ERS (Crone et al., 1998). Local gamma power has been linked to motor performance and motor learning in unimanual tasks (Zich et al., 2021). Conversely, a recent study demonstrated a decrease in gamma ERS after practice, suggesting a potential shift in attentional demands rather than in movement characteristics (Tatti et al., 2023). However, the role of gamma dynamics in bimanual movements, particularly in bimanual movements of varying complexities, remains largely unexplored.

This study was therefore designed with two primary objectives: (1) to investigate beta and gamma activity across unimanual and diverse bimanual movements and (2) to examine how these neural signatures change throughout the learning process. To achieve these goals, we used a motor task we recently developed (Schoenfeld et al., 2021), where participants moved a cursor along an angled path, with each hand controlling movement along orthogonal axes. This approach allowed us to assess different levels of bimanual interaction. We investigated beta ERD and gamma ERS during movement, and beta ERS after movement across different conditions, and tracked how these dynamics changed with practice.

## Materials and Methods

### Participants

Forty-three healthy participants (27 females; mean age ± SD, 24.1 ± 4.2 years) were recruited for this study. All participants were right-handed according to the Edinburgh Handedness Inventory and had normal or corrected-to-normal vision. All fulfilled the following inclusion criteria: (1) no history of neurological or psychiatric disease; (2) no physical disability of the arms or wrists; and (3) no use of drugs affecting the central nervous system. The study was approved by the Central University Research Ethics Committee, University of Oxford (MSD-IDREC-R68649/RE002). All subjects provided written informed consent in accordance with the Declaration of Helsinki.

### Experimental paradigm

Participants were seated upright in the MEGIN TRIUX Neo chair with their heads positioned within the magnetoencephalography (MEG) sensor array. Before the bimanual motor learning task, the force grippers were calibrated for each participant. This calibration required participants to exert maximum effort by squeezing each gripper five times, which established the maximum force for each gripper individually by using the peak maximum relative to the gripper's system offset. During the motor learning task, participants were first familiarized with the task using one orientation street which required equal contributions from both hands to move the cursor diagonally. This was followed by 50 bimanual motor training trials. Each trial consisted of six streets: two unimanual streets at 0 and 90°, two bimanual-equal streets at 45°, and two bimanual-unequal streets at 22.5 and 67.5° (detailed below). Each street started with a 5 s fixation cross at the center of the screen. Following this, a street was displayed whereby the angle of the street indicated the required cursor movement. Participants then started navigating the cursor from the start position (bottom-center of the street) along the ideal path in the middle of the street. If the cursor contacted either side of the street, the cursor was reset to the starting position. Upon reaching the end of the street, there was a 5 s resting interval before the next street was presented. This procedure was repeated for each of the six streets. After completing the sixth street, a final 5 s resting interval was presented, followed by feedback displaying movement time (MT) and accuracy for the full trial.

### Motor learning task

During the bimanual motor learning task, participants were required to guide a cursor along “streets” displayed at different angles on a screen. Specifically, participants held two force grippers, one in each hand, to control the vertical and horizontal movements of the cursor with the left and right hands separately. By squeezing the grippers with varying forces, participants navigated the cursor along an angled street displayed on a screen.

**Figure 1.**
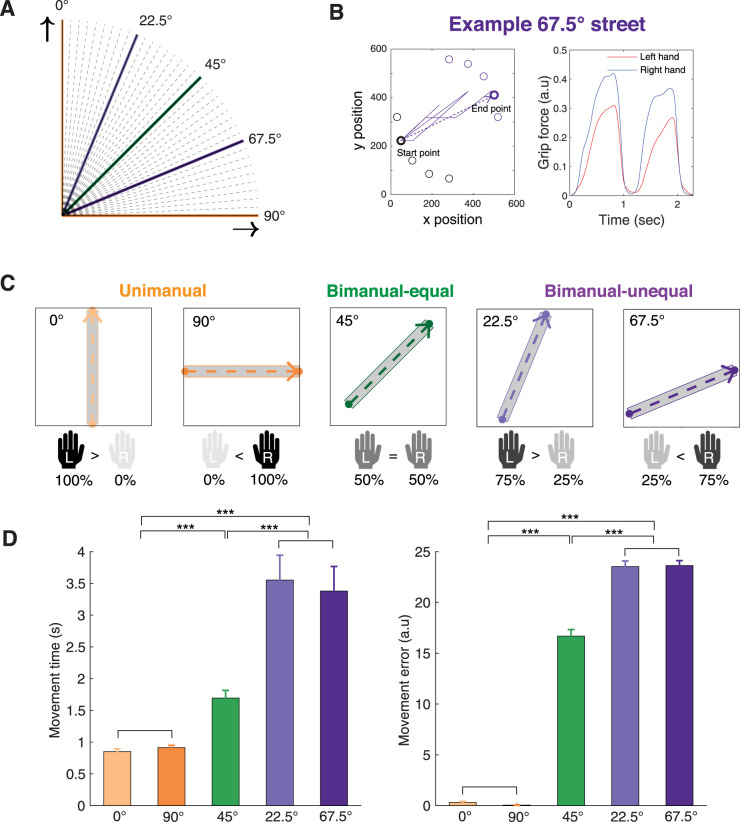
Design of the motor task and behavioral performance. ***A***, Streets are defined at angles of 0, 22.5, 45, 67.5, and 90° to create varying degrees of bimanual hand contribution. The unimanual condition is represented in orange (0 and 90°), the bimanual-equal condition in green (45°), and the bimanual-unequal condition in purple (22.5 and 67.5°). ***B***, Example cursor movement path and hand contribution from a single 67.5° street. The purple lines depict the actual cursor movement path, and the dashed purple line represents the ideal path (left). In this instance, the right hand exerts greater force than the left hand to control the cursor along the 67.5° street (right). ***C***, Hand contribution for streets of different angles. Each hand controls movement along a different axis on the screen: the left hand controls upward movement, and the right hand controls rightward movement. Different angles correspond to specific hand contributions. In the unimanual condition (0 and 90° streets), only one hand is used (left and right hand, respectively). In the bimanual-equal condition (45° street), both hands contribute equally (50%:50%). In the bimanual-unequal condition (22.5 and 67.5° streets), unequal contribution (75%:25% and 25%:75%, respectively) is required. ***D***, Behavioral performance for unimanual, bimanual-equal, and bimanual-unequal conditions. Movement time and error are smaller in unimanual conditions than in bimanual-equal and smaller in bimanual-equal than in bimanual-unequal conditions. Error bars represent the standard error of the mean (SEM). The asterisks indicate significance (***p < 0.001). Figure S4 shows force velocity for unimanual, bimanual-equal, and bimanual-unequal conditions. The correlation between force–velocity and cortical responses is shown in Figure S5.

Each street required specific hand contributions based on its angle (Fig. 1*C*). In the unimanual condition, the vertical (0°) streets were controlled entirely by the left hand, and the horizontal (90°) streets were controlled entirely by the right hand, effectively resulting in hand contributions of 100% from one hand and 0% from the other. In the bimanual-equal condition, the 45° streets required an equal contribution of 50% effort from each hand. In the bimanual-unequal condition, the streets angled at 22.5 and 67.5° required asymmetric contributions, with the 22.5° streets requiring 75% effort from the left hand and 25% from the right and the 67.5° streets requiring 25% effort from the left and 75% from the right (Fig. 1*A*–*C*). To prevent memorization of the order of the streets, the order of the six streets was pseudorandomized across trials, whereby two unimanual streets or two bimanual-equal streets never occurred successively.

Cursor movement required at least 15% of the participant's maximum force. The participant's maximum force moved the cursor by 25 pixels per refresh rate of the monitor (60 Hz). Once the cursor reached the end of a street, participants needed to reduce their grip to <2% of their maximum force to proceed to the fixation cross. This task design was built upon our previous work (Schoenfeld et al., 2021), with the primary modification being an extended time between streets allowing for a postmovement beta ERS and a baseline period for the next street.

### Behavioral data analysis

Movement time and error were calculated for each street. Movement time was defined as the time between movement onset and movement offset. Movement onset was defined as the time when the gripper force exceeded the threshold, calculated as the mean force plus three standard deviations (SD) from the signal during the “rest” period, and was sustained above this level for at least 100 ms (Tan et al., 2014a). Similarly, movement offset was defined as the time before the gripper force fell below this threshold and remained there for at least 100 ms. Error was calculated at each time point by determining the distance of the cursor from the ideal line (the midline of the streets) using Heron's formula. The total error for each street was constituted by the root mean square of errors at all time points (Schoenfeld et al., 2021).

### MEG and MRI data acquisition

Whole-head MEG recordings were acquired in a magnetically shielded room at the Oxford Centre for Human Brain Activity (OHBA) using a 306-channel MEGIN TRIUX Neo system (MEGIN). This system includes 204 planar gradiometers and 102 magnetometers. Head position was monitored using five head position indicator (HPI) coils attached behind the left and right ear lobe, above the right eyebrow, and on the left and right forehead just below the hairline. The locations of the HPI coils, three anatomical fiducial points (nasion and left and right preauricular points), and at least 200 additional points of the head surface were digitized using a Polhemus Fastrak 3D magnetic digitization system (Polhemus) prior to MEG acquisition. Both vertical and horizontal electro-oculograms and electrocardiograms were recorded to monitor eye blinks, horizontal eye movements, and cardiac activity, respectively. MEG data were sampled at 1,000 Hz with a 0.03–330 Hz bandpass filter applied during digitization. Visual stimuli were projected to 31 cm × 55 cm on a screen placed 120 cm in front of the participant using a Panasonic PT-D7700E projector (Panasonic) with a refresh rate of 60 Hz.

T1-weighted structural magnetic resonance imaging (MRI) scans were acquired using a WIN Siemens 3 Tesla Prisma Scanner (Siemens) either at the Centre for Functional Magnetic Resonance Imaging of the Brain or OHBA. The protocol employed a rapid gradient echo (MPRAGE) sequence, which lasted 8 min. The scanning parameters were set as follows: time of repetition, 1,900 ms; time of echo, 3.96 ms; voxel size, 1 mm × 1 mm × 1 mm; field of view, 256 mm × 256 mm (including nose and ears); slice thickness, 1 mm; and 192 slices per slab.

### MEG data analysis

MEG data analyses were conducted using Python 3.10. The MEG data were firstly maxfiltered using the “maxwell_filter” function from MNE-Python for noise removal, bad channel detection, and head movement correction (Gramfort et al., 2013). Subsequent preprocessing steps were performed using the OHBA Software Library (OSL; Quinn et al., 2022). The continuous data were downsampled to 250 Hz to reduce computational demands. A bandpass filter from 0.5 to 100 Hz and notch filters at 50 and 100 Hz were applied. Bad segments were identified by segmenting the data into 500 ms chunks and applying a generalized extreme studentized deviate algorithm at a significance level of 0.1. Independent component analysis (ICA) based on FastICA (Hyvarinen, 1999) decomposition was employed to automatically correct stereotypical heart and eye movement artifacts by correlating the independent components with ECG and EOG channels. Next, any remaining bad channels were interpolated using spherical spline interpolation (Perrin et al., 1989). Two out of 43 participants were excluded from further analysis due to excessive artifacts.

Following preprocessing, source localization was performed to estimate the brain origins of the MEG signal. The MEG data were coregistered with individual T1-weighted MRI images for each participant using headshape points. Three participants lacked individual anatomical scans; for these participants, the MNI152-T1 standard template was used. Prior to beamforming, the data were filtered into two distinct frequency bands: 5–45 Hz primarily for beta power and 60–90 Hz for gamma power. The sensor-level data were then projected onto an 8 mm grid inside the inner skull using a linearly constrained minimum variance beamformer (Van Veen and Buckley, 1988; Westner et al., 2022). A noise covariance matrix used to calculate the beamformer was estimated using the sensor-level data for each subject and regularized to a rank of 60 using principal component analysis. Then, the MEG data were parcellated into 52 anatomically informed regions of interest (Glasser et al., 2016; Kohl et al., 2023). Following this, symmetric orthogonalization was applied to the time courses of these parcels to minimize spatial leakage, and dipole sign flipping was used to align the signs of each parcel time course across subjects (Gohil et al., 2024b).

Next, the data were epoched, aligned to movement onset (from −5 to +10 s relative to movement onset for analyzing beta ERD and gamma ERS) and movement offset (from −2 to +5 s relative to movement offset exclusively for beta ERS analysis). Time–frequency transformation of the MEG data was conducted using the multitaper method, as implemented in MNE-Python. For this analysis, the number of cycles per frequency was dynamically defined to be half of the frequency, and the time–bandwidth product was set at 6. Baseline correction was applied to each epoch within the interval from −1.5 to −1 s relative to movement onset, and the results were converted to decibel (dB) units to normalize power.

**Figure 2.**
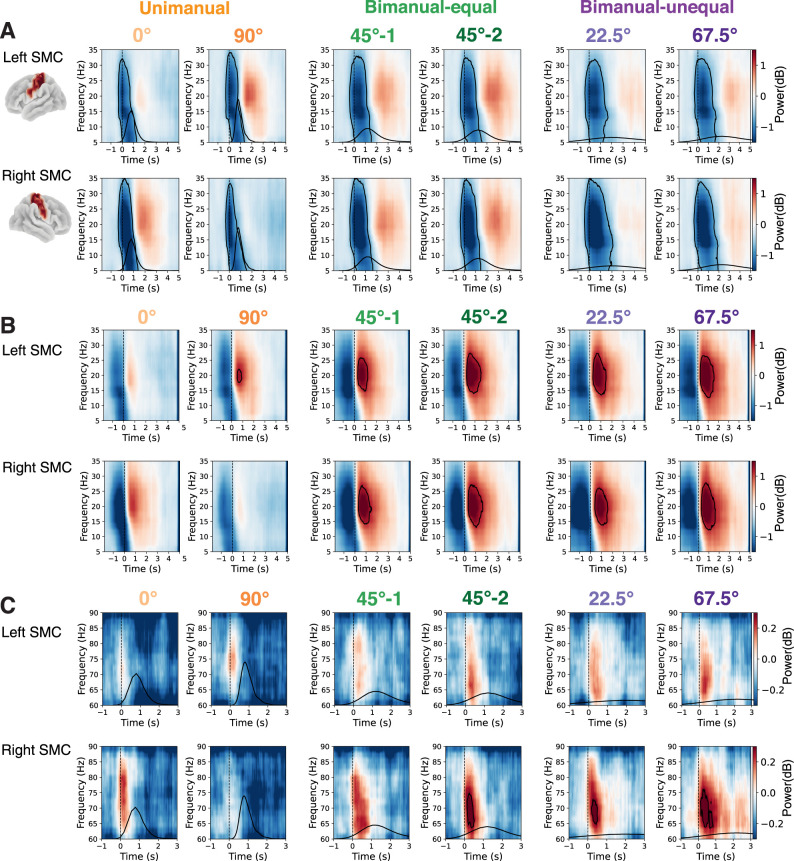
Time–frequency responses to hand movement in the sensorimotor cortex. ***A***, ERD predominantly in the beta (13–30 Hz) band following movement onset in both left and right sensorimotor cortices (SMC) during unimanual and bimanual conditions. Time at 0 s represents movement onset. ***B***, Beta ERS following movement offset, predominantly in the contralateral sensorimotor cortex during the unimanual condition and in both sensorimotor cortices during bimanual conditions. Time at 0 s represents movement offset. ***C***, Gamma (60–90 Hz) ERS following movement onset, primarily in the contralateral sensorimotor cortex during the unimanual condition and in both sensorimotor cortices during bimanual conditions. Time at 0 s represents movement onset. The two columns for the bimanual-equal condition represent the two 45° streets included in each trial. One 45° street from each trial was randomly assigned to one set of 45° streets (45°-1) and the other to a separate set of 45° streets (45°-2). The black outlines in the time–frequency plots indicate significant clusters with *p* < 0.05 by cluster-based permutation test. The black curves represent the distribution of movement times across all subjects.

In the beta band (13–30 Hz), we extracted beta ERD (defined from movement onset to offset) and beta ERS (from movement offset to offset +1 s, Fig. 2). In the gamma band (60–90 Hz), we extracted gamma ERS (from movement onset to onset +0.5 s, Fig. 2). These analyses were specifically focused on the left and right somatosensory and motor cortex areas (Rueda-Delgado et al., 2017).

**Figure 3.**
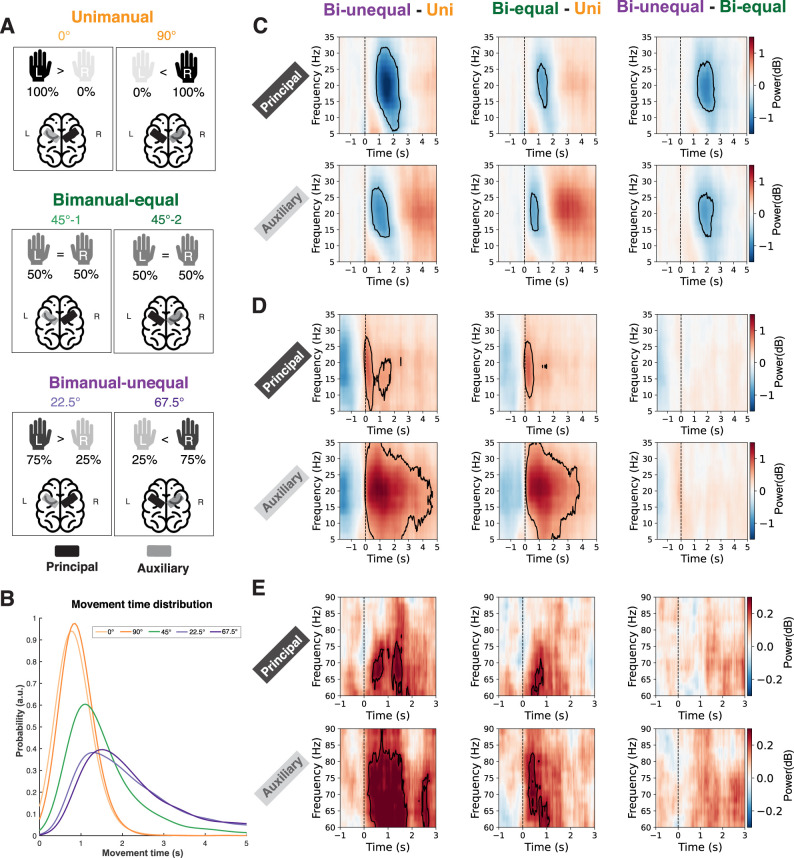
Time–frequency response differences in the sensorimotor cortex for unimanual, bimanual-equal, and bimanual-unequal conditions. ***A***, The black regions indicate the principal sensorimotor cortex (P-SMC), and the gray regions indicate the auxiliary sensorimotor cortex (A-SMC) for the given street. For the unimanual condition, the P-SMC is on the right hemisphere for the 0° street and on the left hemisphere for the 90° street. In the bimanual-equal condition, one 45° street from each trial was randomly assigned to 45°-1 (P-SMC in the right hemisphere) and the other to 45°-2 (P-SMC in the left hemisphere). For the bimanual-unequal condition, the P-SMC corresponds to the hand exerting more force (right hemisphere for the 22.5° street and left hemisphere for the 67.5° street). ***B***, Distribution of movement times across five street angles. ***C***, Greater beta ERD during the movement is observed in two bimanual conditions compared with the unimanual condition (left and middle panels) and in bimanual-unequal compared with bimanual-equal conditions (right panel). ***D***, Greater beta ERS after movement offset is observed in bimanual compared with unimanual conditions (left and middle panels), with no significant difference between bimanual-unequal and bimanual-equal conditions (right panel). ***E***, Greater gamma ERS after movement onset is observed in bimanual compared with unimanual conditions (left and middle panels), with no significant difference between bimanual-unequal and bimanual-equal conditions (right panel). The black outlines indicate significant clusters with *p* < 0.05 by cluster-based permutation test.

For the different movement conditions (unimanual, bimanual-equal, bimanual-unequal), the principal sensorimotor cortex (P-SMC) is defined as the sensorimotor cortex (SMC) contralateral to the hand exerting more or full force, whereas the auxiliary sensorimotor cortex (A-SMC) refers to the SMC ipsilateral to the hand exerting more or full force (Fig. 3*A*). More specifically, in the unimanual condition, the P-SMC is located in the right hemisphere for movements along the 0° street and in the left hemisphere for the 90° street. In the bimanual-equal condition, one 45° street from each trial is randomly assigned to have the right sensorimotor cortex as the P-SMC and the other to have the left sensorimotor cortex as the P-SMC. For the bimanual-unequal condition, the P-SMC is determined by the hand exerting more force, with the right sensorimotor cortex serving as the P-SMC for the 22.5° street and the left sensorimotor cortex serving as the P-SMC for the 67.5° street. Correspondingly, the A-SMC in each condition is the sensorimotor cortex on the opposite side of the P-SMC.

### Statistical analysis

The movement time and errors were compared between conditions using two-tailed paired *t* tests with false discovery rate correction (Fig. 1). To assess the presence of significant ERD and ERS in the time–frequency domain, cluster-based nonparametric permutation tests were performed using 1,000 sign-flipping permutations implemented in the Python toolbox OSL dynamics (Gohil et al., 2024a). These tests compared the observed power against a null hypothesis of zero within specified time windows and frequency ranges as shown in Figure 2. Similarly, cluster-based permutation tests were conducted to identify differences between movement conditions (Fig. 3).

To investigate learning effects, linear mixed-effects models (LMMs) were implemented using the lme4 package in R. Prior to model fitting, the 50 trials were split into five blocks, each containing 10 trials, and trials within blocks were averaged per street angle. Next, the main effect of “block” was tested for each street angle, by including a fixed effect for “block” (five levels, each block corresponding to 10 consecutive trials) and a random intercept for “subject”. To test if the “block” main effect differed across different street angles, a fixed effect for “street angle” (five levels: 0, 22.5, 45, 67.5, and 90°) was included in the model to examine the “block” and “street angle” interaction.

Additionally, to explore the correlations between the behavioral changes (i.e., MT and error) and the neural changes (i.e., changes in beta ERD, beta ERS, and gamma ERS), further LMMs were employed. Specifically, these models included “neural change” as the dependent variable, with fixed effects for “behavioral change” (difference between Blocks 1 and 5) and “street angle,” and a random intercept for “subjects”.

## Results

### Increasing complexity of bimanual movements results in worse task performance

As would be expected, the movement time (MT) for the unimanual conditions was significantly shorter compared with both bimanual-equal (0.88 ± 0.04 s vs 1.69 ± 0.12 s, mean ± SEM, *t*_(40)_ = 8.07, *p* = 1.91 × 10^−9^) and bimanual-unequal conditions (0.88 ± 0.04 s vs 3.47 ± 0.35 s, *t*_(40)_ = 7.80, *p* = 2.21 × 10^−9^; Fig. 1*D*, left). Moreover, MTs were shorter for the bimanual-equal condition than the bimanual-unequal condition (*t*_(40)_ = 5.91, *p* = 6.30 × 10^−7^). Similarly, movement errors were lower in unimanual condition than both the bimanual-equal condition (0.18 ± 0.05 vs 16.69 ± 0.64, *t*_(40)_ = 26.16, *p* = 1.34 × 10^−26^) and the bimanual-unequal condition (0.18 ± 0.05 vs 23.58 ± 0.46, *t*_(40)_ = 52.43, *p* = 5.12 × 10^−38^; Fig. 1*D*, right). Errors in the unimanual condition almost always occurred when subjects used the incorrect hand. There were also lower errors in the bimanual-equal condition than the bimanual-unequal condition (*t*_(40)_ = 12.39, *p* = 2.81 × 10^−15^).

### Increasing complexity of movements results in greater movement-related brain activity

As expected, we observed a significant beta ERD from movement onset for approximately 1.5 s in both the left and right sensorimotor cortices in all conditions (Fig. 2*A*). This was followed by significant beta ERS which persisted for approximately 1–1.5 s from movement offset. The beta ERS was predominantly in the contralateral sensorimotor cortex following the unimanual condition and in both sensorimotor cortices following bimanual conditions (Fig. 2*B*). Gamma power exhibited significant ERS from movement onset for approximately 0.5–1 s, primarily in the contralateral sensorimotor cortex during the unimanual condition and in both sensorimotor cortices during bimanual conditions, though it was more pronounced in the right hemisphere (Fig. 2*C*).

We then went on to compare the movement-related changes in beta and gamma power across the task conditions. There was a significantly greater (i.e., more negative) beta ERD within both the principal and auxiliary sensorimotor cortices (Fig. 3*C*) in both bimanual conditions compared with the unimanual condition. There was also a significantly greater ERD in the bimanual-unequal condition than in the bimanual-equal condition in both SMC.

As might be expected, we observed an extended and more pronounced beta ERS within the auxiliary SMC in the bimanual conditions compared with the unimanual condition (Fig. 3*D*). However, a significantly greater beta ERS was also observed for the bimanual conditions than the unimanual conditions in the principal SMC region, which was engaged in both the unimanual and bimanual conditions. Similarly, the gamma ERS was greater in bimanual conditions than in the unimanual condition, particularly within the auxiliary sensorimotor cortex (Fig. 3*E*). Taken together, these findings suggest that there is increased recruitment of both the primary and auxiliary sensorimotor cortices during bimanual compared with unimanual conditions.

### Motor learning leads to faster and more accurate movements

**Figure 4.**
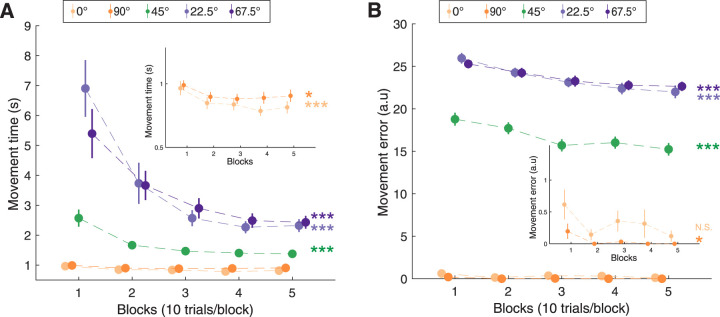
Behavioral performance across blocks for different street angles. ***A***, Movement time significantly decreases across blocks for all street angles. ***B***, Movement error shows a significant decrease across blocks for all street angles except the 0°. Each dot represents the mean performance for a block (10 trials per block) averaged across all subjects, with error bars representing the standard error of the mean across subjects. Statistical significance for the main effect of the factor “block” is shown as: N.S., not significant. *p < 0.05, ***p < 0.001.

Next, we wanted to investigate how behavior changed as participants performed the task. As would be expected, participants’ performance improved with practice, as demonstrated by a significant decrease in MT for each street angle individually [significant main effect of “block” for each street angle: 0° (*χ*² = 23.27, *p* = 1.41 × 10^−6^), 90° (*χ*² = 5.15, *p* = 0.02), 45° (*χ*² = 38.72, *p* = 4.89 × 10^−10^), 22.5° (*χ*² = 46.11, *p* = 1.12 × 10^−11^), and 67.5° (*χ*² = 41.45, *p* = 1.21 × 10^−10^; Fig. 4*A*)].

To investigate the influence of street angle on learning dynamics, we conducted a linear mixed model analysis on MT by further incorporating “street angle” as a fixed effect and revealed a significant interaction between “block” and “street angle” (*χ*² = 86.95, *p* = 5.87 × 10^−18^), indicating that the magnitude of movement time reduction varied as a function of “street angle.” Movement errors significantly decreased during task performance for all street angles except 0° (0°: *χ*² = 2.76, *p* = 0.09; all other street angles, *p* < 0.05; Fig. 4*B*). Similarly to MT, there was also a significant “block” and “street angle” interaction for movement error (*χ*² = 41.15, *p* = 2.50 × 10^−8^).

### Motor learning modulates beta power

**Figure 5.**
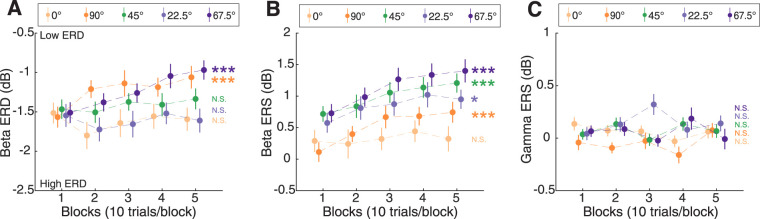
Cortical responses in the principal sensorimotor cortex across blocks for different street angles. ***A***, Beta ERD (from movement onset to offset) in the P-SMC as a function of blocks. Significant changes in beta ERD were observed across blocks for the 90 and 67.5° streets. ***B***, Beta ERS (from movement offset to 1 s after) in the P-SMC as a function of blocks. Beta ERS significantly increased across blocks for all street angles except the 0° street. ***C***, Gamma ERS (from movement onset to 0.5 s after) in the P-SMC as a function of blocks. No significant changes were detected in any street angle. Data points represent the mean power for a block (10 trials per block) averaged across all subjects, and error bars represent the standard error of the mean across subjects. Statistical significance for the main effect of the factor “block” is indicated as: N.S., not significant. *p < 0.05, ***p < 0.001.

Neural responses also changed during task performance. We observed a significantly weaker beta ERD (i.e., the ERD became less negative) in the left, principal SMC as participants learned the task both for 90° (*χ*² = 14.60, *p* = 1.33 × 10^−4^) and 67.5° (*χ*² = 23.99, *p* = 9.67 × 10^−7^; Fig. 5*A*). No significant changes were detected at 0, 22.5, or 45° (*p* > 0.05). The changes in ERD across blocks significantly differed among street angles (“block” and “street angle” interaction, *χ*² = 16.18, *p* = 0.003; Fig. 5*A*).

Beta ERS in the principal SMC significantly increased with task performance in 90° (*χ*² = 20.73, *p* = 5.29 × 10^−6^), 45° (*χ*² = 15.44, *p* = 8.52 × 10^−5^), 22.5° (*χ*² = 6.68, *p* = 0.01), and 67.5° (*χ*² = 21.55, *p* = 3.45 × 10^−6^) streets, with no significant change in 0° streets (*χ*² = 0.41, *p* = 0.52; Fig. 5*B*). There was no significant “block” and “street angle” interaction (*χ*² = 7.82, *p* = 0.10).

No significant block effects were detected in gamma ERS across any conditions in the P-SMC (all *p* > 0.05, Fig. 5*C*). Further analysis of neural responses in A-SMC yielded a similar pattern of results to those observed in the P-SMC (Fig. S1).

### Correlation between changes in task performance and neural responses

Finally, we examined the relationships between the learning-related changes in behavior and changes in neural responses (Block 5 minus Block 1) by using linear mixed models. Given that the unimanual streets frequently contained no errors, our correlation analysis primarily focused on the bimanual conditions, though the overall results remained consistent when including all movement conditions, as detailed in Figure S2.

**Figure 6.**
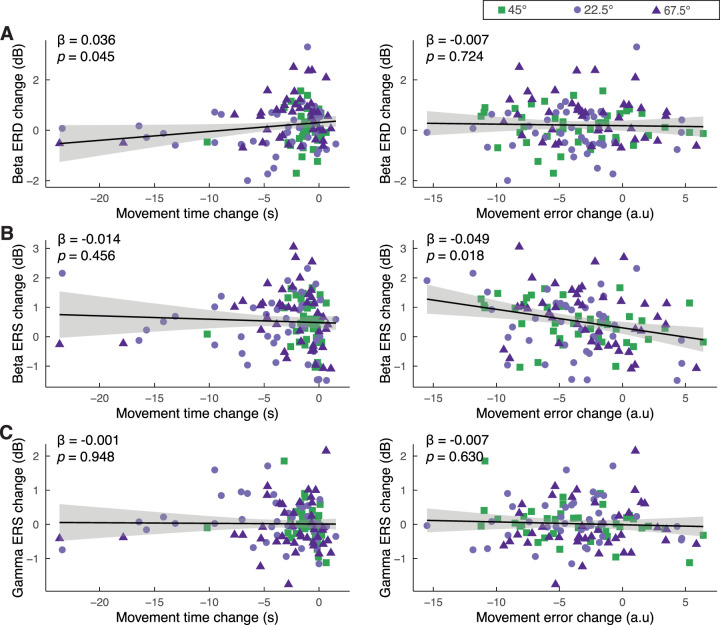
Relationship between learning-related changes in behavior and neural responses. Linear mixed model analysis was used to assess the association between changes in movement performance and changes in neural responses in P-SMC. ***A***–***C***, The changes in beta ERD, beta ERS, and gamma ERS as a function of movement time change (left) and movement error change (right). ***A***, Beta ERD changes exhibit a significant positive relationship with movement time change. No significant relationship is observed with movement error change. ***B***, Beta ERS changes reveal a significant negative relationship with movement error change. No significant relationship is found with movement time change. ***C***, Gamma ERS changes show no significant relationships with either movement time or movement error changes. Data points represent individual trials for the 45, 22.5, and 67.5° streets, colored in green, light purple, and dark purple, respectively. The solid black lines depict the fitted regression lines from the linear mixed models, with shaded areas representing the 95% confidence intervals.

This analysis revealed that learning-related changes in MT showed a trend toward significant correlation with changes in beta ERD in P-SMC (*β* = 0.036, *p* = 0.045; with all conditions included, *β* = 0.030, *p* = 0.089; Fig. S2), indicating that reductions in MT were associated with increasingly negative beta ERD. However, no such association was observed between movement error and beta ERD (*β* = −0.007, *p* = 0.724; Fig. 6*A*). Changes in MT did not correlate with changes in beta ERS (*β* = −0.014, *p* = 0.456). However, a greater reduction in movement errors was associated with an increase in beta ERS (*β* = −0.049, *p* = 0.018), indicating that improvements in task accuracy, reflected by decreased errors, were correlated with increased beta ERS (Fig. 6*B*). Changes in gamma ERS were not related to changes in either MT or movement error (Fig. 6*C*).

### Left and right SMC show differing beta and gamma power

We observed a significant difference in neural responses between left and right SMC. Beta ERD was larger in the right SMC compared with the left SMC, when comparing 0° (right PMC as the P-SMC) and 90° (left PMC as the P-SMC; *χ*² = 40.26, *p* = 2.22 × 10^−10^) and 22.5° (right PMC as the P-SMC) and 67.5° (left PMC as the P-SMC; *χ*² = 35.47, *p* = 2.58 × 10^−9^). The gamma ERS showed a similar hemispheric dominance to the beta ERD, with greater power in the right compared with the left SMC (Fig. 5). This right SMC dominance was also seen in the bimanual-equal condition, where the right SMC exhibits greater beta ERD and larger gamma ERS compared with the left SMC (Fig. S3).

## Discussion

This study demonstrated that unimanual movements were associated with shorter movement times and higher accuracy than bimanual movements. Among the bimanual movements, the unequal movements exhibited longer movement times and lower accuracy than the bimanual-equal movements. On the neural level, we observed significant movement-related activity in terms of beta ERD, beta ERS, and gamma ERS across all movement conditions, with bimanual movements eliciting enhanced responses. As participants practiced the task, their performance improved, reflected by a decrease in movement times and errors. These changes suggest that participants were not merely repeating movements but rather that practice refined their motor skills. In parallel with this improved behavior, we observed an increase in beta ERS during learning, which correlated with the reduction in errors in the task.

### Neural dynamics are modulated during movement

Consistent with previous findings, we observed that beta ERD occurs during movement execution. This reduction in beta power is thought to reflect the release of cortical inhibition and activation of the motor network (Pfurtscheller et al., 1997a; Tinkhauser et al., 2019). Beta ERD is frequently reported bilaterally in electrophysiological (EEG) studies even during unimanual movements (Hummel et al., 2003; Houweling et al., 2008; van Wijk et al., 2012). This contrasts with the more pronounced lateralization seen in hemodynamic responses recorded simultaneously with fMRI-EEG, indicating the complex relation between neurophysiological and hemodynamic responses (Zich et al., 2015). This ipsilateral ERD during unimanual movements may play a role in suppressing unintended mirror-symmetric movement (Diedrichsen et al., 2013; Ames and Churchland, 2019).

Postmovement beta ERS occurs after movement termination and is believed to reflect a prolonged period of increased corticomuscular coherence following voluntary movements (Feige et al., 2000). This increase in beta power is also suggested to be associated with motor plasticity and skill retention (Ghilardi et al., 2021; Peter et al., 2022). Our findings align with previous studies, which report a greater beta ERS in the sensorimotor cortex contralateral to movement (Devos et al., 2006; Jurkiewicz et al., 2006). The small beta ERS in the ipsilateral (left) SMC during left-hand movements and the less obvious beta ERS in the ipsilateral (right) SMC during right-hand movements suggest that the left hemisphere (dominant hemisphere in right-handed individuals) is involved in controlling the left and right hands whereas the right hemisphere (nondominant hemisphere in right-handed individuals) primarily controls left-hand movements (Ziemann and Hallett, 2001).

We observed gamma activity in the 60–90 Hz range coinciding with movement onset and lasting approximately 0.5–1 s, which demonstrates strong lateralization to the SMC contralateral to movement. This unilateral pattern and relatively short time window support the view that gamma ERS has greater spatial and temporal specificity compared with beta activity (Crone et al., 1998; Muthukumaraswamy, 2010).

### Greater task complexity leads to greater movement-related activity

A recent nonhuman primate study using local field potential recordings from the parietal reach region demonstrated task-specific variations in beta power across different bimanual and unimanual tasks (Mooshagian et al., 2021). This study suggested that beta power in both cortices encodes movement-specific information for both hands. In contrast, gamma power likely only encodes contralateral arm movements (Mooshagian et al., 2021). Correspondingly, our analysis revealed that beta ERD varies across different motor tasks in both hemispheres, supporting its role in encoding task complexity. However, gamma ERS was significantly activated in the A-SMC during bimanual tasks, but not in unimanual movements, resulting in a pronounced difference primarily in the A-SMC between bimanual and unimanual movements.

fMRI studies have shown that incompatible bimanual tasks involving amplitude or direction interference elicit stronger activation in the parietopremotor network than fully compatible tasks (Wenderoth et al., 2004, 2005). Together with our MEG results, these findings indicate that greater task complexity with increased interlimb interference enhances cortical activity. Compared to previous studies showing interhemispheric inhibition or increased interhemispheric coherence (Andres et al., 1999; Serrien et al., 2003; Fling and Seidler, 2012), our study revealed enhanced local beta and gamma activity during bimanual movements compared with unimanual ones. However, we cannot directly link local brain activities to interhemispheric communication. Future research is required to understand how local brain activities and interhemispheric communication interact and contribute to bimanual coordination.

### Increases in beta ERS correlate with error reduction during learning

We demonstrated significant increases in postmovement beta ERS across the task, except at the 0° street angle, supporting the relationship between increases in learning-related beta ERS and error reduction. Previous studies have demonstrated that postmovement beta ERS in the sensorimotor cortex is associated with learning processes (Tan et al., 2014b, 2016; Torrecillos et al., 2015). However, the precise functional role of beta ERS remains unclear.

According to early theories, beta ERS serves an active inhibitory function by “clearing out” the motor program after movement execution and resetting the neural network to its baseline state (Kilavik et al., 2013; Schmidt et al., 2019). Recent studies, however, propose that the role of beta ERS extends beyond simply reflecting an inactive or inhibited cortical state. Beta ERS has been shown to relate to movement error (Tan et al., 2014a, 2016; Torrecillos et al., 2015). Consistent with this, our finding that the learning-related increase in beta ERS correlates with a learning-related decrease in error suggests that beta ERS may be involved in the comparison between planned and executed movements. Another potential explanation is that the ERS increase may reflect improvements in interlimb coordination during learning, potentially involving interhemispheric coupling (Kajal et al., 2017; Iwama et al., 2022; Van Hoornweder et al., 2022). Notably, these changes were observed during early learning but may diminish with extended practice as performance reaches asymptotic levels.

### Beta ERD is reduced during learning, but only in the left, principal SMC

Unlike beta ERS, we did not observe consistent changes in beta ERD during practice across different movement conditions: only conditions in which the left sensorimotor cortex was principally involved showed a less negative deflection in ERD. The role of beta ERD in learning is not consistent across the previous literature. For example, one study reports a practice-induced strengthening in beta ERD (i.e., a more negative deflection; Zich et al., 2018). In contrast, other studies find no significant differences in sensorimotor beta ERD during learning (van der Cruijsen et al., 2021; Pavlova et al., 2023), challenging the notion that beta ERD reflects learning-related neural adaptations.

All our participants were right-handed; hence, the left SMC is in the dominant hemisphere in all participants. It is not possible, therefore, to unpick whether the differences between the pattern of learning-related changes in the left and right SMC are due to intrinsic hemispheric differences or rather reflect the dominant hemisphere.

### Gamma ERS does not change with learning

Our study observed no significant changes in gamma ERS during practice in any movement conditions, suggesting that gamma ERS may not play a critical role in motor learning quantified by reduced movement time and reduced error. This observation appears to contradict the view that gamma activity plays a prokinetic role in movement and motor skill acquisition (Muthuraman et al., 2020; Zich et al., 2021). For instance, studies using 75 Hz or theta–gamma coupling transcranial alternating current stimulation have shown relationships between 75 Hz activity and skill acquisition (Nowak et al., 2018; Akkad et al., 2021). However, they studied thumb abduction acceleration rather than the movement time and error used here; it may therefore be that 75 Hz activity more directly reflects force modulation than other aspects of motor performance.

### Task considerations

The differences in neural responses between conditions might result from variations in movement times, rather than differences in bimanual coordination per se. However, the temporal specificity of significant differences in neural dynamics makes this explanation less likely. For example, the gamma ERS clusters (Fig. 3*E*) align closely with movement onset. If the differences in gamma ERS across movement conditions were caused by the extended movement time, we would expect the cluster to appear later, during the prolonged movement phase, rather than at the movement onset when both unimanual and bimanual movements share a similar duration. This temporal specificity is in contrast with the variability and delayed onset of significant beta ERD clusters when comparing different task conditions, indicating that movement time may influence beta ERD (Fig. 3*C*). Furthermore, correlation analyses reveal a correlation between changes in movement time and changes in beta ERD but not with changes in gamma or beta ERS, supporting that the observed differences in beta ERD, but not beta ERS or gamma ERS, between conditions could be affected by movement time. In addition, we further compared beta ERS and gamma ERS across conditions by taking MT into account. No significant main effect of MT was observed.

In conclusion, this study enhances our understanding of the neural mechanisms distinct to unimanual and bimanual movements, revealing differential responses of beta and gamma activities to these varying task complexities. Furthermore, our findings advance insights into the neural underpinnings of motor learning, particularly emphasizing the functional significance of beta ERS. This suggests that specific neural rhythms can be strategically targeted to optimize motor learning and recovery processes. Our study focused on intrahemispheric dynamics, but future work could explore interhemispheric coupling to illustrate how cross-cortical communication interacts with local dynamics during bimanual movements. Furthermore, given the complexity of the task, it is important to explore brain regions beyond the SMC, particularly those involved in cognitive control.

## Data Availability

The datasets and code will be shared via the Medical Research Council Brain Network Dynamics Unit at the University of Oxford data sharing platform on https://data.mrc.ox.ac.uk/.
